# 

**Published:** 1872-11

**Authors:** 


*Virginia State Dental Association.*—The regular annual meeting of the Virginia State Dental Association will be held in the city of Richmond, on Wednesday, Oct. 30th, 1872.

We urge all to attend, as a duty they owe to their patients. Those attending can visit the State Fair which will be held at the same time; and thus half fare will be secured.

